# Insights Into Unveiling a Potential Role of Tertiary Lymphoid Structures in Metastasis

**DOI:** 10.3389/fmolb.2021.661516

**Published:** 2021-09-08

**Authors:** Rami Mustapha, Kenrick Ng, James Monypenny, Tony Ng

**Affiliations:** ^1^ Richard Dimbleby Laboratory of Cancer Research, School of Cancer and Pharmaceutical Sciences, King’s College London, Guy’s Medical School Campus, London, United Kingdom; ^2^ Cancer Research UK King’s Health Partners Centre, London, United Kingdom; ^3^ UCL Cancer Institute, University College London, London, United Kingdom; ^4^ Department of Medical Oncology, University College Hospitals NHS Foundation Trust, London, United Kingdom; ^5^ Cancer Research UK City of London Centre, London, United Kingdom

**Keywords:** TLS, metastasis, premetastatic niche, high endothelial venule (HEV), lymphangiogeneis, tissue stress, interstitial fluid pressure, tertiary lymphoid organs

## Abstract

Tertiary lymphoid structures (TLSs) develop in non-lymphatic tissue in chronic inflammation and cancer. TLS can mature to lymph node (LN) like structures with germinal centers and associated vasculature. TLS neogenesis in cancer is highly varied and tissue dependent. The role of TLS in adaptive antitumor immunity is of great interest. However, data also show that TLS can play a role in cancer metastasis. The importance of lymphatics in cancer distant metastasis is clear yet the precise detail of how various immunosurveillance mechanisms interplay within TLS and/or draining LN is still under investigation. As part of the tumor lymphatics, TLS vasculature can provide alternative routes for the establishment of the pre-metastatic niche and cancer dissemination. The nature of the cytokine and chemokine signature at the heart of TLS induction can be key in determining the success of antitumor immunity or in promoting cancer invasiveness. Understanding the biochemical and biomechanical factors underlying TLS formation and the resulting impact on the primary tumor will be key in deciphering cancer metastasis and in the development of the next generation of cancer immunotherapeutics.

## Introduction

Tertiary lymphoid structures (TLSs) develop postnatally in non-lymphoid tissue following a prolonged inflammatory state. The structure of a TLS can vary from a simple aggregation of lymphocytes and myeloid cells to a more organized structure resembling a lymph node (LN) without a capsule (Ruddle, 1999). In the literature, TLSs have been described by various nomenclatures including ectopic lymphoid structures and tertiary lymphoid organs; in this review we will refer to them as TLS. The structure of TLSs depends on their level of maturity. They can display high endothelial venules (HEV)-like vessels to connect with the blood circulation, lymphatic vessels to connect with the lymphatic circulation, a follicular dendritic cell network for antigen presentation to B cells, and functional germinal centers that include T/B cell spatial compartmentalization for activation and clonal expansion.

TLSs are considered lymphoid organs owing to their resemblance to LNs, mainly with regards to their cellular content organization, vasculature and most importantly their function. However, despite their similarities, there are key differences between LNs and TLSs mainly with regards to the cellular interactions leading to their development. By definition, TLSs develop postnatally in a variety of non-lymphoid tissues and organs. While LN formation is genetically pre-programmed, TLSs are formed after accumulation of inflammatory signals produced by tissue stromal cells and infiltrating lymphocytes following a repeated, unresolved and prolonged immune response. The exact molecular processes and cellular interactions governing the formation of a TLS and the function of the stromal component of the TLS are highly variable, depending on tissue origin and disease pathology (Ruddle, 2020) (Marinkovic and Marinkovic, 2020).

**FIGURE 1:**
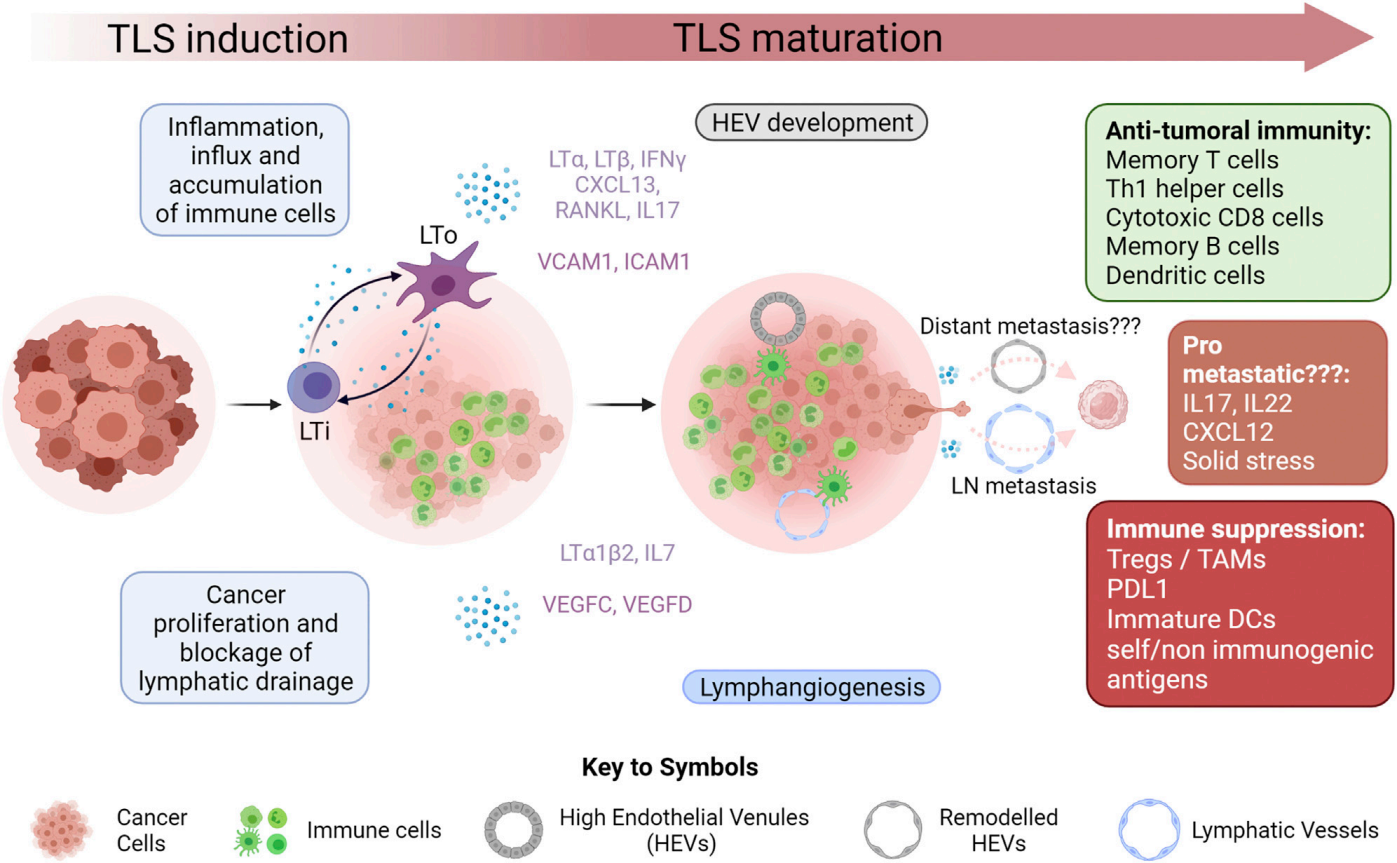
Tertiary Lymphoid Structure Development in Cancer Inflammation within the microenvironment of growing tumors results in increased infiltration of immune cells. This, coupled with the effect of rapidly proliferating cancer cells, often results in blockage of draining lymphatic vasculature, leading to an accumulation of immune cells and initiation of tertiary lymphoid structure (TLS) formation within the tumor or at its boundary. A key step in this initiation is the interaction of lymphoid tissue inducers (LTi) with lymphoid tissue organizers (LTo). The nature of these cells is highly variable in cancer. This interaction results in the secretion of a myriad of cytokines favoring the maturation of the TLS by recruiting various immune cells through newly created lymphatic vessels and high endothelial venules (HEVs). While this maturation often results in a strong anti-tumoral immune response, it has also been shown to occasionally favor immunological tolerance mainly by negatively affecting antigen presentation machinery and by recruiting immune suppressive cells such as regulatory T cells (Tregs) and tumor associated macrophages (TAMs). This new vasculature has a potential role in tumor metastasis by acting as conduits for metastasizing cancer cells. Lymphangiogenesis has been correlated with increase lymph node (LN) metastasis. Whether or not HEVs are linked to distant metastasis still remains to be definitively proven. The cytokine rich environment of the TLS while being favorable to anti-tumor immunity has also been shown to have pro-metastatic potential in certain cases. Eventually TLSs in cancer can organize into more defined LN like structures with T cell zones and B cell follicles but this was not depicted in this figure.

In this review, we will focus on TLSs and the neogenesis of their associated vasculature. We will highlight recent breakthroughs in our understanding of LN and distant metastasis and will provide insight into how TLS involvement in cancer metastasis can address some unanswered questions regarding metastasis initiation, and how TLS associated vessels can provide potential routes for tumor cell transport to the circulation. A graphical summary of the proposed role of TLS in metastasis is shown in (Figure 1).

### Metastasis

Metastasis remains the leading cause of death in cancer, accounting for up to two thirds of deaths in patients with solid tumors (Dillekas et al., 2019). Metastasis is a multi-step process. First, it requires malignant primary tumor cells to gain an invasive phenotype and dissociate from the primary tumor. These cells then need to gain access to the systemic circulation either via lymphatic or blood vessels, seed the distant tissue and then proliferate to form a new malignancy at that site - all while evading the immune system.

Our understanding of distant metastasis has historically been derived from *in-vivo* models. Recent data obtained from histopathological analysis of cancer tissue coupled with cutting edge genetic sequencing of primary tumors, LN metastases and distant metastases have further elucidated the process of metastasis in patients. When discussing metastasis, it is important to highlight the distinction between LN metastasis/invasion and distant metastasis/metastasis via the lymphatics which we suggest can depend on TLS. The conventional viewpoint is that distant metastases are derived from either the hematogenous or lymphatic routes (Wong and Hynes, 2006). Distant metastasis is far less likely to occur than LN metastasis, a fact that has been attributed to the ease of access to the LN via the rich lymphatic vasculature structures surrounding many solid tumors.

However, there appears to be mechanistic bottlenecks hindering distant metastasis. By using genetic sequencing of multi-region biopsies in colorectal cancer, Reiter et al., found far higher levels of inter and intra-lesion heterogeneity in LN metastases compared to distant metastases where lesions were genetically similar to each other and typically of the same clonal origin (Reiter et al., 2020). There are two potential explanations for this: firstly, lymph nodes drain multiclonal tumor cells at any given point, therefore giving rise to a greater degree of intra-lesion heterogeneity. This is in direct contrast to distant metastases, where outgrowth may be derived by a single successful clone. Alternatively, it may suggest that fewer primary tumor clones are capable of seeding distant organs, indicating a unique and more intense selective pressure on cancer cells for seeding distant metastases. If this holds true, the major bottleneck is not the ability of a malignant cell to exit the tumor into the circulation but rather the ability of this cell to safely travel the blood circulation and establish a colony in a new organ (Vanharanta and Massague, 2013). For example, oxidative stress in the blood and viscera of seeded organs is known to limit metastasis in melanoma (Piskounova et al., 2015).

The fact that LN metastasis is more common than distant metastasis has led to the assumption that it acts as a precursor for distant metastasis; yet this is still heavily debated. Clinically, overall survival was not improved by axillary LN dissection in addition to chemotherapy and radiotherapy in breast cancer and melanoma patients (Giuliano et al., 2011; Faries et al., 2017). However, this does not mean that lymphatic metastasis is inconsequential to patient survival as LN involvement is one of the biggest factors in staging cancer. The significance of the ability of LN invading cancer cells to seed distant metastasis was proven in several *in-vivo* models. Pereira et al. (2018), used three different photoconvertible tumor cell lines; mammary carcinoma, squamous cell carcinoma and melanoma; to prove that cancer cells can exit the LN, join the systemic blood circulation and metastasize into the lungs. The authors also found that tumor cells were more likely to associate with HEVs of the LN, even invading the basement membrane on their way out of the LN rather than the lymphatic vessels. Interestingly lung metastasis in this model did still occur when the LN was pre-emptively excised before induction of the primary tumor albeit at far lower levels indicating that there are other possible routes of tumor metastasis (Pereira et al., 2018). Similar conclusions on the route of metastasis from the LN to the distant site were independently drawn by Brown et al. (2018), who used intra-lymphatic micro-infusions to place breast cancer cells directly in the subcapsular sinus of the LN in mice (Brown et al., 2018). They showed that these cells follow the same path observed in breast cancer patients as they migrated within the LN before eventually exiting via the HEVs, to join the circulation and seed distant metastases. While HEVs may serve as exit points for cancer cells, they may also serve as entry ports for incoming lymphocytes into the LNs, potentially creating a ‘contraflow’ between cancer and immune cells. Interestingly, they further showed that this happened well before the tumor cells had travelled through the efferent lymphatics and drained into the circulation at the thoracic duct and the subclavian vein, which meant that the lymphatics were not the likely route of this transport. In a seminal article, Ubellacker et al. showed that the lymphatics act as a key factor in endowing melanoma cells with the ability to resist oxidative stress. In this work, they demonstrated that both murine and human cells could far more efficiently establish distant metastasis when injected intra-lymphatically than intravenously. Melanoma cells suffered far lower levels of ferroptosis-induced oxidative stress in the lymph than the blood as they incorporated oleic acid in triacylglycerols of ApoB + vesicles into their plasma membrane which even permitted them to resist oxidative stress in the distant organ and circulation after exiting the lymphatics (Ubellacker et al., 2020).

While this *in-vivo* data showed a clear role for lymphatics and in particular via LNs in establishing distant metastasis, LNs alone cannot tell the whole story of distant metastasis. Using whole exome sequencing of primary tumors, sentinel LN metastases and distant metastases, phylogenetic analysis from multiple cancers has revealed that the origin of distant metastases is actually complicated (Sanborn et al., 2015). In multiple cancers; only a fraction of the distant metastases were of the same clonal origin as the LN metastases. In 213 samples from 17 patients with colorectal cancer only 35 percent of distant metastases shared a common sub-clonal origin as the LN metastases, which is expected for a cancer that particularly favors hepatic metastasis via the portal vein (Naxerova et al., 2017). However, what is surprising are the findings of Venet et al., who showed that in estrogen receptor positive breast cancer, 75 percent of the distant metastasis did not share a common clonal origin with the axillary LN (Venet et al., 2020).

The simplest explanation for these data is that the majority of metastases are seeded by cells that disseminate via the hematogenous route. However, this assumption ignores the inhospitable nature of the circulatory system to tumor cells, either via oxidative stress or via sheer physical forces (Follain et al., 2020). A plausible alternative route for distant metastasis would be the extra nodal lymphatic route via the TLS. It has been proven that tumoral growth in the draining LN can lead to obstruction of the afferent lymphatics. This is followed by lymphangiogenesis (the growth of *de novo* lymphatic vessels) in a new draining LN which in theory might be the source of distant metastasis (Leijte et al., 2009; Kwon and Sevick-Muraca, 2010). Whether lymphangiogenesis is TLS driven remains to be confirmed. On the other hand, it is entirely possible to imagine that the TLS and its associated HEVs capable of directly linking with the blood are paving the way for cancer spread. Indeed clues hinting to this are starting to appear in clinical data from breast cancer patients. Histopathological analysis on postsurgical resection of the primary tumor on a cohort of 290 patients revealed a weak association between the presence of intratumoral TLS and lymph node involvement (Figenschau et al., 2015).

In the next section we will discuss the different stages of TLS development from formation to maintenance and maturation. We will then highlight how these stages can either directly promote distant metastasis by inducing tumor invasiveness or indirectly by providing vasculature connections between the tumor and the systemic circulation.

### Tertiary Lymphoid Structure formation and maintenance: Possible impact in metastasis?

TLS formation is often described using the same two-cell model governing LN organogenesis. This model is based on interactions between two cell types, namely lymphoid tissue inducers (LTis) and lymphoid tissue organizers (LTos). Using gene knockout murine models that fail to develop LNs, scientists have identified these cells as distinct cell types. Classical LTis are a fetal liver derived subgroup of innate lymphoid cells 3 (ILC3) expressing nuclear retinoid orphan receptor γt (RORγt), IL-7 receptor and inhibitor of differentiation 2 (Id2). In LN organogenesis, RANKL and IL7 expression by LTos activates LTis which then express lymphotoxin α (LTα) and lymphotoxin β (LTβ2) to activate VCAM1 and ICAM1 expression by LTos leading to chemokine production and angiogenesis. This initiates immune cell recruitment leading to aggregation, organization and formation of a LN (Onder and Ludewig, 2018). When comparing LNs to TLS, there are key considerations regarding LTis and LTos. As TLSs are very similar to LNs, their cellular equivalents of lymphoid tissue inducers (LTis) and lymphoid tissue organizers (LTos) have been extensively researched (Gago da Graca et al., 2021). Firstly, unlike LNs, TLSs can develop in different tissues with different stromal cells which means that the nature and type of the LTo cell within the TLS will be tissue specific. Moreover, classical LTis do not seem to be crucial in TLS development, as surrogate cells take over that role in mouse models deficient for RORγt and Id2 (Marinkovic et al., 2006; Lochner et al., 2011; Marinkovic and Marinkovic, 2020). It seems that the ability to induce TLS formation does not depend on a specific cell type but rather a cell’s ability to secrete specific cytokines in response to appropriate stimuli. These cytokines, by interacting with tissue specific stromal cells, lay the grounds for increasing immune cell infiltration, organization, establishment of HEVs and even lymphangiogenesis. For example, TLSs developed in inflamed organs in mice deficient for RORγ or Id2, and which were therefore lacking LTis in the intestines and the thyroid, respectively (Marinkovic et al., 2006). This induction was dependent on interactions between myeloid cells and T cells and the secretion of TNFα or CCL21 (Furtado et al., 2014). However, natural cytotocity receptor (NCR)-positive ILC3 with LTi capabilities were identified in the periphery of TLSs in non-small-cell-lung-cancer (NSCLC). These cells expressed RORγt, secreted high amounts of cytokines including IL22, IL17, TNFα and LTα1β2, and even induced expression of ICAM-1 and VCAM-1 on mesenchymal cells (Carrega et al., 2015). Th17 cells are another example of cells that have been shown to act as LTis in multiple settings (Pikor et al., 2015). Th17 cells have a lot in common with classical LTis mainly regarding RORγt expression, production of IL17 and IL22 and expression of LTα1β2 (Grogan and Ouyang, 2012). Numerous tissue specific chemokine and cytokine signatures are crucial for TLS development and influx of immune cells. These signatures from various pathologies have recently been described elsewhere (Gago da Graca et al., 2021). IL17 and IL22 are two key cytokines in fibroblast priming for an LTo role, yet both of these cytokines are known to have a pro-metastatic effect. IL17 was shown to be highly expressed in TLSs from multiple tissue origins and in different pathologies (Peters et al., 2011). Interestingly, in bronchus associated lymphoid tissue, IL17 induced iBALT formation in an LTi independent but LTα dependent manner. IL17 was also shown to induce CXCL12 production by stromal cells in the other TLSs (Fleige et al., 2014). CXCL12 only binds CXCR4 which is highly expressed on multiple tumor cells, especially in triple negative breast cancer. The CXCL12/CXCR4 pathway has numerous pro-metastatic effects. Not only does it increase epithelial mesenchymal transition (EMT) and the motility and invasion of breast cancer cells, it also recruits endothelial progenitor cells and induces angiogenesis (Zielinska and Katanaev, 2020). IL17 was also shown to promote metastasis in lung cancer by directly promoting EMT in lung cancer. *In-vivo* blocking of IL17 slowed cancer progression and decreased the metastatic load (Salazar et al., 2020). In addition, IL17-mediated signaling may preclude responses to immunotherapy in mismatch repair-proficient (MMRp) colorectal cancer patients (Llosa et al., 2019). IL22 is another Th17 related cytokine that is highly expressed in different TLSs. The origin of IL22 in TLSs varied depending on the tissue and pathology. In salivary gland inflammation it was expressed by γ/δ T cells whereas it was expressed by ILC3 in *C. rodentium* infection in the colon (Ota et al., 2011; Barone et al., 2015). Regardless of the origin, IL22 seems to play a role in TLS maturation rather than initiation. IL22 in cancer was shown to promote hepatocellular carcinoma proliferation and metastatic potential via activation of the STAT3 pathway (Jiang et al., 2011). IL22 was also shown to increase matrix metalloprotease expression in breast cancer thus increasing their invasiveness (Markota et al., 2018; Kim et al., 2020). Finally, in triple negative and HER2+ breast cancer patients, the amount of LTi or ILC3 cells in primary tumors was shown to correlate with the level of LN metastasis in the same patients (Irshad et al., 2017).

### Tertiary Lymphoid Structure maturation and associated vasculature: highways for metastasis?

A possible role for TLS in metastasis would mainly stem from the fact that as the TLS develops it promotes ectopic lymphangiogenesis and HEV formation. This *de novo* vasculature has been shown to play a key role in anti-tumoral immunity in most cancers but this does not exclude a potential role in acting as a route of spread for tumor cells to reach either the draining LN or the circulation. In the following parts, we will focus on the role of lymph node associated vasculature in metastatic spread which has been well documented and attempt to link it with data on TLS associated *de novo* vascular formation and metastasis which is currently less evident.

### High Endothelial Venules:

HEVs are anatomically distinct venules. They are a post capillary connection to the blood circulation found in secondary lymphoid organs with the exception of the spleen (Girard et al., 2012). They provide a route of flow for the entry of peripherally circulating lymphocytes to the LN (Pfeiffer et al., 2008. HEVs are formed of specialized endothelial cells called high endothelial cells (HECs) that are characterized by their unique plump, cuboidal morphology. HECs express classic endothelial cell markers such as CD31 and VE-cadherin along with specific functional markers like peripheral node addressin (PNAd) which binds CD62L (L-selectin) on lymphocytes ultimately resulting in lymphocyte extravasation into the LN parenchyma. While the bulk of research on HEVs was obtained from LNs, it is now accepted that HEVs do develop ectopically in tissue during chronic inflammation and cancer.

In cancer, increased HEV density is an independent positive predictive indicator in various solid tumors (Table 1), and usually associated with immune cell infiltration. MECA79 staining by immunohistochemistry which identifies PNAd positive vessels with distinct morphological characteristics is the traditional method for assessing HEV density in tumors. In a prospective analysis of 127 primary breast carcinomas, HEV density correlated with increased infiltration of CD4^+^ Th1 helper cells, cytotoxic CD8^+^ T cells, memory T cells and B cells identified by flow cytometry analysis of primary tumors. Interestingly HEV density did not correlate with the density of classical blood vessels identified by CD34^+^ endothelial cells suggesting different pathways of angiogenesis between HEVs and classical blood vessels (Martinet et al., 2011). HEVs were detected in two thirds of a 225 primary tumor melanoma cohort. High HEV density correlated with less tumor cell invasion, decreased thickness and signs of regression. HEV density was associated with increased infiltrating CD4^+^ and CD8^+^ T cells, DC Lamp^+^ dendritic cells but not Foxp3^+^ Tregs in the vicinity of the HEV rich area assessed by tissue staining. Transcriptomic analysis on a sub cohort of 14 samples identified overexpression of T cell recruiting cytokines and chemokines including (CCL5, CXCL9, CXCL10, CXCL11, CCL19, CCL21 and CXCL13) as well as recruitment related receptors (CXCR3, CCR7 and CD62L) (Martinet et al., 2012). Similarly HEVs were found in a cohorts of 75 primary tumor of advanced stage oral squamous cell carcinoma where their presence correlated with better prognosis. Interestingly they were more abundant in low grade disease (Wirsing et al., 2016). A recent study on colorectal cancer (CRC) further correlated TLS associated tumoral HEV density with mismatch repair. Higher HEV density along with higher B and T lymphocyte infiltration was more likely to be found in hereditary microsatellite instable (MSI) CRC, compared to patients with sporadic MSI CRC and microsatellite stable (MSS) disease. HEV density also correlated with PD1/PDL1 expression and was increased in B2M mutant CRC compared to wildtype clearly showing that tumoral antigenicity is a key factor in HEV formation (Pfuderer et al., 2019). In stark contrast, in a separate study on 62 CRC primary tumors, TLS associated HEVs were mostly found on the invasive margin and did not correlate with intra tumoral lymphocyte invasion. Higher HEV density was found in MSS but not MSI tumors and it was associated with more advanced disease (Bento et al., 2015). The contradicting data obtained in these two studies indicates that the role of TLS and their associated HEVs could extend beyond recruitment of immune cells.

**TABLE 1:** High endothelial venules as a prognostic and predictive indicator in solid tumors.

Author	Tumor type	N, patients	Key findings
Martinet et al. (2011)	Invasive Breast Cancer	146	Higher densities of tumor HEVs associated with a lower risk of relapse and positively correlated with longer metastasis-free, disease-free and overall survival rates
Martinet et al. (2012)	Melanoma	225	High densities of tumor HEVs associated with tumor regression, low Clark level of invasion and thin `Breslow thickness
Wirsing et al. (2016)	Oral Squamous Cell Carcinoma	75	High density of tumor HEV associated with smaller tumours (T1-T2) and lower risk of disease-specific death
Pfuderer et al. (2019)	Colorectal cancer	83 (MSI = 48, MSS = 35)	Higher density of HEV in MSI compared to MSS colorectal cancers, with implications on the potential of HEV as an additional predictive biomarker for use of immune checkpoint inhibitors in colorectal cancer

Legend: HEV = High Endothelial Venules, MSS = Microsatellite Stable, MSI = Microsatellite Unstable.

Development of HEVs in cancer is not fully understood with the majority of the data on ectopic HEV neogenesis being obtained from autoimmune and inflammatory conditions. Nonetheless, HEV formation is considered to be a key step in TLS maturation allowing for increased influx of immune cells, mainly naïve T and B lymphocytes (Gago da Graca et al., 2021). Similar to the induction of the formation of LNs, that of TLSs depends on a Lymphoid tissue inducers (LTis) or an LTi like cells accumulating at the future TLS site and interacting with lymphoid tissue organizers (LTos); local stromal cells expressing lymphotoxin β2 receptor (LTβR). This interaction involves multiple pathways and cytokines including LTα and LTβ, CXCL13, RANKL and IL7 as well as LTos producing VCAM1 and ICAM1 which results in HEV formation. However, unlike HEVS in LNs, TLS-associated HEVs rely on TNF signaling to develop, are independent of lymphotoxin β receptor-mediated signaling and crucially do not express CCL21 (Colbeck et al., 2017). Data from subcutaneous and intraperitoneal models of murine melanoma and lung cancer have revealed a key role for LTα3 and IFNγ signaling in TLS associated HEV formation. LTβR blocking only disrupted PNAD expression on LN HEVS; resulting in decreased immune infiltration; but did not affect PNAD nor CCL21 levels of tumoral HEVS. In this model, using a combination of various gene deficient mice, the authors identified two cytokines that played a major role in PNAD^+^ CCL21^+^ HEV neogenesis. IFNγ produced by either CD8 T cells or NK cells induced CCL21 expression in tumoral HEVs by fibroblasts and endothelial cells but did not affect PNAD expression. Moreover, LTα3 but not TNFα engagement with TNFR1/2 on endothelial cells induced PNAD expression (Peske et al., 2015). In a separate and contradictory study in human breast cancer, overexpression of LTβ but not LTβR, LIGHT nor LTα was found in primary tumors with high HEV density. Its levels correlated with levels of CCL21, CCL19 and CXCL13. DC-lamp^+^ dendritic cells were found to be the source of LTβ, as they colocalized with HEVs in primary tumors and retrospectively correlated with disease free survival in a cohort of 146 patients with invasive breast cancer (Martinet et al., 2013). The formation of HEVs does usually correlate with the influx of immune cells and anti-tumoral immunity yet the exact function of HEVs seems to be tumor specific because a potential role for immunosuppression related to HEVs has been described. For example, high Treg infiltration was observed in high density HEV tumors (Martinet et al., 2013). In melanoma, CCL21 expression resulted in the formation of a Treg rich TLS which disrupted antigen presentation and hindered T cell mediated anti-tumoral immunity (Shields et al., 2010; Joshi et al., 2015). Treg depletion resulted in increased CD8^+^ T cell infiltration and CD8 T cell driven HEV development. HEV development has been recently reviewed in details (Blanchard and Girard, 2021).

Currently, few studies have directly correlated HEV formation with distant metastasis. Evidence on the potential role of HEVs in LN metastasis is found in oral and pharyngeal carcinoma (OPSCC). A study on 64 OPSCC primary tumors correlated an increase in tumoral ectopic HEV density with LN metastasis. In this study, HEC proliferation was accompanied by HEV remodeling where tumor cells were more likely to be associated with remodeled HEVs. The authors postulated a role for L-selectin in HEV mediated LN metastasis showing that the use of CD62L neutralizing antibodies decreased tumor cell LN adhesion (Shen et al., 2014). The exact anatomic connection between an ectopic HEV; which is by definition an afferent venule connection from the circulation to the TLS, and the draining LN, remains to be fully understood. At this point in time, hard data showing cancer cells exiting the primary tumor site via such HEVs are lacking but this possibility should not be excluded and merits further study.

### Lymphatic Vasculature

Lymphatic vessels are specialized, blind ended vasculature that allow the unidirectional flow of liquids, lipids, other soluble components and cells away from the tissue to the LN and eventually back into the blood circulation. They play a crucial role in maintaining interstitial fluid (ISF) homeostasis, lipid transport and immunity. The major component of these vessels are lymphatic endothelial cells (LECs), which are a distinct population of endothelial cells expressing prospero-related homeobox 1 (PROX1), podoplanin (GP38 which is also expressed by fibroblasts), CD31 (which is also expressed by classical blood vessel endothelial cells) and vascular endothelial growth factor (VEGF) receptor–3 (VEGFR-3). There are different levels of lymphatic vessels, the initial highly branched blind ended lymphatic capillaries are formed of hyaluronan receptor 1 (LYVE1) expressing LECs with discontinuous junctions and lacking a basement membrane. This results in a direct connection to the tissue microenvironment allowing easy uptake of ISF, macromolecules and immune cells (Baluk et al., 2007). The lymph is carried to the collecting vessels, that portray continuous cell-cell junctions, a basement membrane, pericytes and lymphatic smooth muscle cells (LMSCs) whose contractions, along with arterial pulsation, drive the movement of the lymph through LNs to larger lymphatic ducts, eventually emptying into the blood circulation via the thoracic duct or right lymphatic duct. Retrograde lymph movement and blood reflux in the lymphatic vessels is prevented by intraluminal valves in collecting vessels and lymphovenous valves at lymphaticovenous junctions. These larger vessels and collecting vessels do not usually express LYVE1 (Petrova and Koh, 2020). LYVE1 interaction with hyaluronic acid (HA) on dendritic cells was shown to be crucial for their adhesion to, and migration through, lymphatic capillaries towards draining LNs and initiation of antigen presentation (Johnson et al., 2017). Lymphangiogenesis happens both during embryonic development and in adult life in differentiated tissue. The majority of embryonic LECs are transdifferentiated from venous precursors but non-venous progenitors have been shown to play a role in supplementing the lymphatic vasculature in different organs such as the intestines (Escobedo and Oliver, 2016). Prox1 is not only a key transcription factor in the differentiations of LECs but also in the maintenance of their identity during both embryonic development and during ectopic adult lymphangiogenesis (Johnson et al., 2008; Cho et al., 2019). TLS development is also accompanied by lymphangiogenesis which has been very well described in immune diseases (Antonioli et al., 2020). A viral based model to induce TLS formation in salivary glands that mimics Sjo¨gren’s syndrome showed that lymphatic vasculature development in the TLS, similar to that in SLO was dependant on IL-7, LTα1β2 and immune cell accumulation (Nayar et al., 2016). In a model for CCL21 induced thyroiditis in which TLS develops in the thyroid, dendritic cell (DC) recruitment by LTα expressing CD4 T cells was shown to be key for generation of new lymphatic vasculature (Muniz et al., 2011). DCs have also been shown to be important in the maintenance of influenza induced TLS in the lungs (GeurtsvanKessel et al., 2009).

### Lymphangiogenesis and Metastasis

Multiple *in-vitro* and pre-clinical *in-vivo* studies have demonstrated an upregulation in the production of VEGFC/D in solid tumors from different cellular sources and correlated that with higher lymphangiogenesis and LN/distant metastases (Mandriota et al., 2001; Skobe et al., 2001; Stacker et al., 2001; Hirakawa et al., 2007). This was further confirmed because the inhibition of the VEGFC pathway, either by blocking antibodies or by VEGFC traps, resulted in decreased metastases (Lin et al., 2005; Gogineni et al., 2013). However, clinical data on the role of VEGFR signaling in metastasis have mainly focused on correlating expression levels with lymphatic vessel density, LN metastasis or prognosis in multiple cancer types (Dadras et al., 2003; Yokoyama et al., 2003; Juttner et al., 2006; Shida et al., 2006; Li et al., 2009; Tacconi et al., 2015). Clinical trials targeting lymphangiogenesis have started. ALTER 0303 was a placebo controlled trial for anlotinib which is a pan receptor tyrosine kinase inhibitor. In a patient cohort of advanced lung adenocarcinoma patients, lymphatic vessel density correlated with disease progression and poor prognosis. Moreover, drug treatment was shown to suppress lymphangiogenesis in 66 patients (Han et al., 2018; Qin et al., 2020). A phase one clinical study using LY3022856/IMC-3C5 which is an anti VEGFR3 monoclonal antibody was reported in 2016, with modest anti-tumor activity (Saif et al., 2016).

### Lymphatic Vessels: Immunological Tolerance Allows Safe Travel for Cancer

The role of lymphatic vessels, particularly those of LECs, in immunomodulation is an area of great interest. Given that primed and activated immune cells travel constantly through these vessels, a certain level of suppression is understandable to ensure peripheral tolerance. The bulk of the research has however focused on tolerance at the level of the LN LECs. One key function of LN LECs is their ability to cross-present via MHCI endogenously expressed tissue-restricted self-antigens. This coupled with their low levels of co-stimulatory molecules, CD80, CD86 and ICOSL and their expression of suppressive molecules including PDL1, LAG3, IDO, TFGβ and NO results in suppression of autoreactive CD8^+^ T cells (Tewalt et al., 2012; Card et al., 2014). Moreover, under steady state conditions, LN LECs can actively uptake foreign antigens in the lymph, process them and cross present them to CD8^+^ T cells indicating that they play a clear role in tolerance to foreign antigens (Hirosue et al., 2014). Regarding CD4^+^ T cell tolerance, LN LECs were not efficient in loading peptides on to MHCII due their lack of H2-M, however, cross presentation of loaded MHCII complexes from DCs has been proven to play a role in antigen specific CD4 T cell tolerance (Dubrot et al., 2014; Rouhani et al., 2015). Whether or not TLS associated LECs possess similar immunomodulatory capabilities is a heavily researched area and the implications of that in cancer immunology are still not understood.

In colorectal cancer, the VEGFC/VEGFR3 axis was shown to be upregulated in human primary cancers and to contribute to metastasis and disease progression on multiple levels. VEGFR3 was not only found on LECs but also on tumor associated macrophages (TAMs). Tie-2 expressing/hi macrophages, which may be related to alternatively activated (M2) subpopulation of TAMs can interact with lymphatic endothelial cells and cause the latter to contract, thereby facilitating tumor cell lymphatic metastasis to LNs (Evans et al., 2019). VEGFC was shown to increase neogenesis of lymphatic vessels with increased permeability and higher number of infiltrating cancer cells. Moreover, it was shown to recruit and activate immunosuppression in TAMs (Tacconi et al., 2015; Tacconi et al., 2019). The role of this axis in immunosuppression was also shown in over expression models and was dependent on prostaglandin synthase expressing LECs (Christiansen et al., 2016).

In models of syngeneic melanoma and breast cancer, IFNγ commonly overexpressed in the tumoral microenvironment was shown to upregulate PDL1 on tumor associated LECs which consequently inhibited T cell mediated anti-tumor immunity (Dieterich et al., 2017). Dermal LECs can express ICAMs in the context of TNFα mediated inflammation which binds to Mac1 (CD11b/CD18 heterodimer) on DCs assisting their migration through the lymphatics but also suppressing their differentiation (Johnson et al., 2006; Podgrabinska et al., 2009). These data provide insight into the potential role of immunosuppression around and within tumor lymphatic vasculature. Such inhibitory signals could be key in allowing safe travel for a cancer cell outside the tumor via TLS associated *de novo* vasculature and into the circulation.

### Blood Vessels vs Lymphatic Vessels vs Ectopic High Endothelial Venules as Preferred Route of Metastatic Spread?

The route that cancer cells follow from the tumor to the draining LN is still under investigation. Available data suggest that HEVs and lymphatics associated with TLS can play a key role in this transport. One indication that travel via lymphatics is more likely than transport via blood vessels can be derived from clinical trials using anti-angiogenic drugs in which an increase in the metastatic potential of the primary tumor was observed (Ebos, 2015). These drugs selectively inhibited angiogenesis of blood vessels but not of HEVs nor lymphatic vessels. Data obtained from longitudinal imaging of primary tumor vasculature and LN metastasis from multiple mouse cancer models show that newly formed blood vessels are not the likely route for invading tumor cells as antiangiogenic drugs do not affect nodal metastasis. These data were also confirmed by immunohistochemistry staining of clinical tumor samples from head and neck and colorectal cancer patients that had a positive response to anti-angiogenic therapy in which nodal metastasis did not corelate with blood vasculature density (Jeong et al., 2015). It was later shown that the anti-angiogenic drug sunitinib, which inhibits multiple receptor tyrosine kinases, induces lymphangiogenesis in renal cell carcinoma (RCC) by inducing VEGFC expression which was associated with increased LN metastasis (Dufies et al., 2017). However, these findings do not deny a potential role for HEVs in this transit. In fact, the research on angiogenesis and nodal metastasis discussed above does not discriminate between HEVs and normal vasculature. The studies used a pan endothelial marker CD31 and as such a potential correlation between HEVs and nodal metastasis was not assessed. In fact, an *in-vivo* mouse study on three spontaneous mouse cancer models for breast cancer, pancreatic cancer and glioblastoma found that VEGF inhibition when combined with anti-PDL1 therapy was successful only when it induced HEV neogenesis in the breast and pancreatic cancer model but not in the glioblastoma model. Whether the increase in T cell infiltration was due to the HEV induction or whether the inflammatory flood induced by immune checkpoint inhibition coupled with anti-angiogenic factors that triggered the HEV remains to be seen. While these studies did not look at metastasis, they do demonstrate that ectopic HEV neogenesis in primary tumors relies on different molecular cues than classical angiogenesis (Allen et al., 2017). Essentially, with our current understanding of tumor vasculature, it is impossible to single out one single culprit in this transit and more research is required in untangling all these potential mechanisms to help push the development of metastasis-targeting cancer therapy.

### Tertiary Lymphoid Structure at the Metastatic Site; There Is Still Much to Learn

To fully address the potential role of TLS in metastasis, we need to look at TLS beyond the site of the primary tumor and focus on the actual site of metastasis. There is limited data on the presence and function of TLS at metastatic sites and even less data directly comparing these with TLS at the primary tumor and metastases. To date, TLS at the metastatic site have been mostly linked to some form of anti-tumor response (Sautes-Fridman et al., 2019). A study on breast cancer metastasis showed that TLS development was dependent on location of the metastasis with TLS being found in the liver and lung but not in the brain and ovary (Lee et al., 2019). However, a separate study looking at lung metastasis from either CRC or RCC showed that the nature of TLS immune infiltrate was influenced by the origin of the primary tumor. The presence of TLSs rich in CD8^+^ T cells and DC-LAMP^+^ dendritic cells correlated with better survival in CRC yet worse survival in RCC (Remark et al., 2013). Another study on TLSs in lung metastasis of CRC similarly found that the presence of TLS did not correlate with survival. However, the nature of the T cell subsets in the TLS did (Schweiger et al., 2016). Two studies on liver metastasis again from CRC correlated the presence of TLS at the invasive margin of the metastasis but not within it with better prognosis (Meshcheryakova et al., 2014; Ahmed and Halama, 2020). TLS in peritoneal metastasis of high-grade serous ovarian cancer had a functional anti-tumor B cell response signature which was enhanced by chemotherapy (Montfort et al., 2017). These studies show that TLS can form in the metastatic site. Their formation seems to have a favorable impact on prognosis yet is still highly varied and dependent on both the origin of the primary tumor and the location of the metastatic site. One interesting study used a vascular remodeling agent made of LIGHT and a vascular targeting peptide known to induce HEV formation and correct leaky vasculature to prevent lung metastasis and the premetastatic niche (He et al., 2020). This possibly indicates that the degree of TLS maturation at the site of potential metastasis can have differential effects on the ability of tumor cells to seed that site.

### Premetastatic Niche and Lymphatic Vasculature

The simplest definition of the premetastatic niche is the changes occurring in the site of the future metastasis rendering its microenvironment more hospitable and receptive to tumorigenesis. These changes are triggered by factors derived from the primary tumor or the primary tumor stroma (Doglioni et al., 2019). In a breast cancer model, we previously showed that macrophages associated with LECs at the site of the lung metastasis in a β4 integrin/TGFβ1 dependent manner which resulted in lymphatic remodeling facilitating tumor spread (Evans et al., 2019). VEGFC was shown to induce lymphangiogenesis in the sentinel LN even before seeding by tumor cells in models of chemically induced skin carcinogenesis (Hirakawa et al., 2007). A separate study on melanoma using whole body lymphangiogenesis imaging via VEGFR3 as a surrogate marker revealed that exosomal midkine was key for lymphatic expansion in the metastatic site. Midkine which is a heparin-binding factor induced LEC proliferation via paracrine effects of the mTOR pathway on VEGFR3 expression (Zhou et al., 2016). This and other work show that lymphatic vasculature at the site of the future metastasis is key for the circulating malignant cell’s ability to seed and establish a tumor (Bieniasz-Krzywiec et al., 2019). The importance of lymphangiogenesis at the metastatic site was recently demonstrated in a study on melanoma patients with lung metastasis (Ma et al., 2018). Lymphatics may potentially even play a much more key role in the spread of tumor derived pre-metastatic niche drivers than previously believed. Components of lymphatic exudates from melanoma patients obtained during lymphadenectomy were analyzed and compared to plasma from the same patients and to lymphatic exudates from healthy donors. Interestingly, despite having an overall lower protein content than plasma, lymph was more highly enriched in melanoma related factors. Those factors included extra cellular vesicles (carrying miRNAs), cytokines, chemokines and proteins implicated in immunosuppression, metastasis and the establishment of pre-metastatic niche. These included, LDH, S100B, S100A8, CSF-1, Galectin-3, MMMP-2, IL6, IL8, TNFα, IL1β and IL10. These factors were higher in metastatic melanoma patients than non-metastatic ones. Of these S100A9 has been demonstrated to be a target for imaging the pre-metastatic niche *in vivo* (Eisenblaetter et al., 2017). Of special interest, VEGFC was highly enriched in the lymph particularly when compared with VEGFD which was enriched in the plasma. The lymph also contained higher levels of undifferentiated T cells, MDSCs and CD163^+^ monocytes. This data shows that lymphatics are a clear route of spread of tumor derived factors related to progression and establishment of the pre-metastatic niche (Broggi et al., 2019).

### Investigating the Potential Role of Intratumoral Mechanical Forces in Promoting Tertiary Lymphoid Structure Neogenesis and Tumoral Metastasis in Certain Cancers

Distinct physical changes in the tissue often accompany tumorigenesis. These include increases in solid stresses, interstitial fluid pressure and stiffness (Nia et al., 2020). Elevated solid stress arises from the accumulation of cells and aberrant matrix deposits pushing against the surrounding healthy tissue. These cells can be rapidly proliferating cancer cells, or rapidly infiltrating immune cells (Nia et al., 2016). The forces resulting from accumulation of cancer cells can compress intra-tumoral vasculature; both blood and lymphatic, decreasing flow rates and preventing adequate drainage (Padera et al., 2004). Decreased fluid drainage coupled with an increase in tissue fluids leaking from newly formed hyper permeable blood vessels results in increased interstitial fluid pressure (IFP) which creates sheer stress on the tumoral and stromal cells in the microenvironment (Swartz and Lund, 2012; Sefidgar et al., 2015). Mechanical stresses, both solid and sheer, have pro-metastatic role by directly inducing cancer cell invasiveness and indirectly by inducing TLS related lymphangiogenesis. Glioma cell expressed matrix metalloproteases MMP1 and MMP2 as a direct response to increases in fluid stress (Qazi et al., 2011). Sheer fluid mechanosensing in cancer cells was dependent of CD44 and its interaction with hyaluronic acid in the glycocalyx around tumor cells (Qazi et al., 2013). Using a combination of patient derived primary cells and *in-vivo* models, Choi et al. showed that sheer fluid stress induced EMT and higher tumorgenicity in breast cancer (Choi et al., 2019). Solid stress was also separately shown to induce migration and cytoskeleton remodeling in breast cancer cells (Tse et al., 2012). Other work on the role of mechanical stress in cancer progression has been described in detail elsewhere (Northcott et al., 2018).

One of the key signals in TLS induction is the accumulation of immune cells at the site of the inflammation, and this alone suggest a role for mechanosensing in TLS formation (Ruddle, 2020). The effect of mechanical stresses on TLS formation was recently observed in a study of lung injury in adult mice. The study showed that increases in IFP following impairment of lymphatic drainage could induce TLS formation (Reed et al., 2019). One possible mechanism explaining mechanical stress induced lymphoid neogenesis is the presence of mechano-sensors on LECs. High interstitial fluid pressure was shown to induce embryonic lymphangiogenesis in a process involving LEC proliferation and elongation following VEGFR3 activation. Integrin-linked kinase (ILK)/β1 integrin complex was shown to be responsible for sensing changes in mechanical stress. Mechanical force on the LEC released β1 integrin from the complex, which in turn interacted with VEGFR3, facilitating its phosphorylation and lymphangiogenesis. ILK knockout resulted in uncontrolled lymphatic growth in a β1 integrin dependent manner. Conditional ILK knockout in adult mice resulted in LEC proliferation in both vascular and avascular tissue. *In-vitro* mechanical stretching of primary human adult LECs led to dissociation of (ILK)/β1 integrin complex and VEGFR3 phosphorylation. These results show a molecular mechanism through which mechanosensing of tumoral mechanical stress can play a key part in ectopic lymphangiogenesis (Planas-Paz et al., 2012; Urner et al., 2019). Ectopic lymphangiogenesis and increased lymphatic flow was shown to precede melanoma metastasis (Harrell et al., 2007). Inhibition of integrin α4β1 suppressed lymphangiogenesis and controlled metastasis in multiple *in-vivo* spontaneous and orthotopic models including spontaneous breast cancer (Polyoma middle T) and orthotopic models for several cancer (Garmy-Susini et al., 2010). High ISF pressure is also responsible for a key process of draining lymphatic rerouting in metastatic cancer that can explain discrepancies in phylogenetic origins of distant metastasis compared to LN metastasis. Proulx et al. (2013), used *in-vivo* imaging of tumor lymphatics in models for melanoma and breast cancer to show that lymphatic obstruction occurs following metastasis to the draining LN, which leads to an increase in tumoral ISF pressure resulting in lymphangiogenesis and rerouting of tumor draining lymph to a different LN (Proulx et al., 2013). Evidence on the obstruction of draining LN as a function of tumor burden was also found in breast cancer patients (Zhou et al., 2016). Decreased lymphatic drainage leading to an increase in ISF pressure can be attributed to impaired contractions of lymphatic endothelium smooth muscle cells (LESMC). NO secreted by tumor associated Gr1^+^ myeloid cells expressing inducible nitrous oxide synthase iNOS in the vicinity of lymphatic capillaries was shown to inhibit LESMC contraction in multiple tumor models (Liao et al., 2019). The data above show that LECs can sense an increase in ISF pressure and that TLS formation and lymphangiogenesis act in tandem to restore fluid equilibrium in the tissue. This results in increased vasculature which, as described above, can facilitate tumor escape by providing travel routes for tumor cells or by helping create the pre-metastatic niche (Follain et al., 2020). Mechanical stresses in the tumor have an impact on carcinogenesis which is worthy of further investigation to better understand their role in TLS formation and metastasis. The development of tools which can non-invasively measure changes in tumor stiffness or ISF pressure will be invaluable not only in future cancer research but also in patient therapy. Magnetic resonance elastography (MRE), a variation of magnetic resonance imaging, is capable of non-invasively measuring tissue biomechanics. Fovargue et al. (2020), have recently shown that MRE can be used to non-invasively measure tumoral pressure (Fovargue et al., 2020).

## Conclusion and Perspectives

In conclusion, the role of TLS in cancer immunity and immunotherapy has been of major interest lately as multiple studies in cancer patients correlated the presence of mature, germinal center containing TLSs with response to immunotherapy (Cabrita et al., 2020; Helmink et al., 2020). While literature reviews have focused on the role of TLS in generating anti-tumor immunity (Sautes-Fridman et al., 2016; Sautes-Fridman et al., 2019), in this review we highlight literature data on the function of TLS in metastasis. Even though the role of lymphatics in metastatic spread is now undeniable, recent data clearly show that the draining LN is not responsible for seeding the entirety of distant metastases. After the primary tumor invades the draining LN, it proliferates and effectively blocks lymphatic drainage from the primary tumor. This results in an increase of ISF pressure which, in parallel with increases in solid stresses, promotes tumor EMT and lymphangiogenesis. This occurs concomitantly with an influx of T cells, B cells and myeloid cells interacting with the stromal fibroblasts creating early TLSs. Based on the types of immune cells interacting in the early stages of TLS formation this can create a TH17/ILC3 cytokine/chemokine signature which even further favors tumor invasiveness. As the TLS matures, it promotes the formation of HEVs and lymphatic vasculature to further increase immune cell recruitment. Yet this *de novo* vasculature has been shown to possess immunoregulatory functions ultimately enabling safe passage of cancer cells to the peripheral circulation. Furthermore, lymphatics also carry tumor derived factors to assist in the formation of the premetastatic niche. Better understanding of key pathways in TLS induction can help the development of immune-therapeutics tailored at skewing TLSs towards a clear anti-tumoral immune function. This, along with imaging techniques to measure tissue stresses in cancer will lead to better diagnosis of early metastasis and also potentially biomarkers for precision immunotherapy in cancer patients.
